# Achieving similar root microbiota composition in neighbouring plants through airborne signalling

**DOI:** 10.1038/s41396-020-00759-z

**Published:** 2020-09-24

**Authors:** Hyun Gi Kong, Geun Cheol Song, Hee-Jung Sim, Choong-Min Ryu

**Affiliations:** 1 Molecular Phytobacteriology Laboratory, Infectious Disease Research Center, KRIBB, Daejeon, 34141 South Korea; 2 Crop Protection Division, National Institute of Agricultural Sciences, Rural Development Administration, Wanju-gun, 54875 South Korea; 3 Plant Nutrition Laboratory, Industrial Microbiology Center, CJ, Suwon, 16509 South Korea; 4 Environmental Chemistry Research Group, Korea Institute of Toxicology (KIT), 17 Jegok-gil, Munsan-eup, Jinju, 52834 South Korea

**Keywords:** Microbial ecology, Soil microbiology

## Abstract

The ability to recognize and respond to environmental signals is essential for plants. In response to environmental changes, the status of a plant is transmitted to other plants in the form of signals such as volatiles. Root-associated bacteria trigger the release of plant volatile organic compounds (VOCs). However, the impact of VOCs on the rhizosphere microbial community of neighbouring plants is not well understood. Here, we investigated the effect of VOCs on the rhizosphere microbial community of tomato plants inoculated with a plant growth-promoting rhizobacterium *Bacillus amyloliquefaciens* strain GB03 and that of their neighbouring plants. Interestingly, high similarity (up to 69%) was detected in the rhizosphere microbial communities of the inoculated and neighbouring plants. Leaves of the tomato plant treated with strain GB03-released β-caryophyllene as a signature VOC, which elicited the release of a large amount of salicylic acid (SA) in the root exudates of a neighbouring tomato seedling. The exposure of tomato leaves to β-caryophyllene resulted in the secretion of SA from the root. Our results demonstrate for the first time that the composition of the rhizosphere microbiota in surrounding plants is synchronized through aerial signals from plants.

## Introduction

Volatile organic compounds (VOCs) represent one of the many plant-to-plant signalling systems [1]. Given their diffusivity, VOCs function as important signalling molecules that transmit the status of a plant to adjacent and distant plants [1]. Plant VOCs are released in response to biotic stresses, such as herbivore and pathogen attack, and abiotic stresses caused by environmental fluctuations [2–4]. Signals emitted from damaged plants can cause “plant-to-plant communication”, which induces defence of nearby plants. Volatile substances such as C6 fatty acids, isoprenoids (mostly terpenes), methyl salicylate (MeSA) and indoles are known as herbivore-induced plant volatiles (HIPVs) [5]. Recently, a study proposed that plant volatiles could be divided into two distinct categories, depending on the type of biotic stress inducer: microbe-induced plant volatiles (MIPVs) and HIPVs [6].

Studies show that infection of plants by microbial pathogens elicits the release of MIPVs. For example, *Cucumber mosaic virus* increased the total emissions of volatiles from *Cucurbita pepo* plants [7]. *Barley yellow dwarf luteovirus* increased the amount of (*Z*)-3-hexenyl acetate released by wheat (*Triticum aestivum* L.) plants [8]. Potato plants infected by *Potato leaf roll virus* showed increased release of six volatile substances [9]. In addition to viruses, bacterial pathogens also trigger the release of plant volatiles. A virulent strain of the bacterial pathogen *Pseudomonas syringae* pv. tomato induced the release of β-ionone and α-farnesene from *Arabidopsis thaliana* (*Arabidopsis*) plants, while infection by an avirulent strain of this pathogen increased MeSA release, suggesting that volatile material composition varies with the virulence of the pathogen [10]. During the infection of apple (*Malus pumila* var. *domestica*) plants by *Erwinia amylovora* or *P. syringae*, VOCs such as hexenal isomers and 1,2-propanediol were released. In addition, activation and signalling of salicylic acid (SA) synthesis has been found in healthy plants exposed to volatiles produced by neighbouring plants infected with *E. amylovora* [11]. In the case of kiwifruit infected with *P. syringae* pv. actinidiae, the release of hexane and decane increased [12]. Plants are also affected by the production of volatiles induced by pathogenic fungi. Resistant wheat varieties inoculated with the stripe rust fungal pathogen *Puccinia triticina* mainly released β-ocimene, and treatment with volatiles significantly decreased disease severity in susceptible wheat varieties and lima bean (*Phaseolus lunatus* L.) plants [13, 14]. In addition, volatiles including nonanal and MeSA of lima bean plants released upon treatment with a chemical trigger, benzothiadiazole (BTH), induced resistance to the bacterial pathogen *P. syringae* pv syringae among neighbouring plants [15].

Interestingly, plant-beneficial microbes including nitrogen-fixing rhizobia, mycorrhiza and plant growth-promoting rhizobacteria (PGPR) can also modify the plant volatile, MIPV profile to induce plant defence [16–20]. Pretreatment of faba bean (*Vicia faba* L.) plants with the symbiotic arbuscular mycorrhizal fungi reduces the release of sesquiterpenes [18]. The rhizobia-colonized lima bean plants showed that indole represents the agent of the repellent effects at jasmonic acid (JA)-induced VOCs [17]. PGPR, free-living root-associated bacteria, have been studied since the last century with the aim to increase plant growth and productivity [21, 22]. Studies show that PGPR promote plant growth via bacterial determinants that induce the production and phosphate dissolution of plant hormones such as indole 3-acetic acid, gibberellic acid, JA, SA, and cytokinin [23–25]. However, most studies investigating the mechanism of plant growth promotion by PGPR generally focus on the direct physiological response of treated plants, and the interaction between plants and bacteria [6, 26]. In a recent study, *Pseudomonas putida* KT2440, a PGPR, induced the production of indole and β-caryophyllene volatiles in maize plants [19]. Similarly, another PGPR, *Pseudomonas simiae* WCS417r, in the rhizosphere of *Arabidopsis*, enhanced the attraction of parasitoid, *Microplitis mediators*, against the leaf-chewing insect, *Mamestra brassicae* [20]. These studies suggest that PGPR elicit innate immune responses in plants by triggering the release of plant volatiles and their transmission from one plant to another.

To date, only a few studies have investigated the role of PGPR-elicited release of MIPVs on plant-to-plant communication [19, 20]. There has been no study on the microbiome aspects of the rhizosphere of volatile-emitter plants treated with PGPR and that of receiver (neighbouring) plants. Root exudates such as sugars, organic acids, secondary metabolites and complex mucus-like polymers play a critical role in re-shaping the root microbiota [24]. The composition of root exudates varies with the plant genotype, developmental stage and stress [27–29]. Therefore, root exudates serve as the main physiological tool employed by plants to control the microbial community in the rhizosphere, depending on environmental conditions [29–33]. Root exudates such as JA and SA affect the rhizosphere microbial community. In *Arabidopsis*, deletion of JA and SA biosynthesis genes altered the rhizosphere microbial community of mutant plants compared with that of wild-type plants [24, 25]. However, it is unknown whether the release and function of MIPVs, following the application of biological control agents and PGPR on the root system, affect the composition of the rhizosphere microbiota of neighbouring plants.

It is well known that introduction of a new PGPR into the plant rhizosphere modulates the indigenous rhizosphere microbiota. However, the effect of the newly introduced PGPR on the rhizosphere microbiota of neighbouring plants has not been investigated to date. Two spatially separated plants can communicate through airborne signalling of VOCs. In this study, we adapted the MIPV concept and hypothesized that PGPR application on one plant affects the rhizosphere microbiota of the neighbouring plant through MIPV. To test this hypothesis, we employed metagenome analysis to examine the rhizosphere microbiota of a tomato (*Solanum lycopersicum* L.) plant treated with *Bacillus amyloliquefaciens* GB03, which is a model PGPR, and that of a neighbouring (spatially separated) tomato plant. We identified β-caryophyllene as a MIPV in the emitter tomato seedling and SA as a critical root exudate of the neighbouring plant. Intriguingly, the rhizosphere microbiota diversity of the PGPR-treated emitter plant was highly similar to that of its neighbouring receiver plant. Thus, our data demonstrate for the first time that an MIPV derived from a PGPR can serve as a driving force to modulate the rhizosphere microbiota of spatially distant plants.

## Materials and methods

### Experimental design

**Fig. 1:**
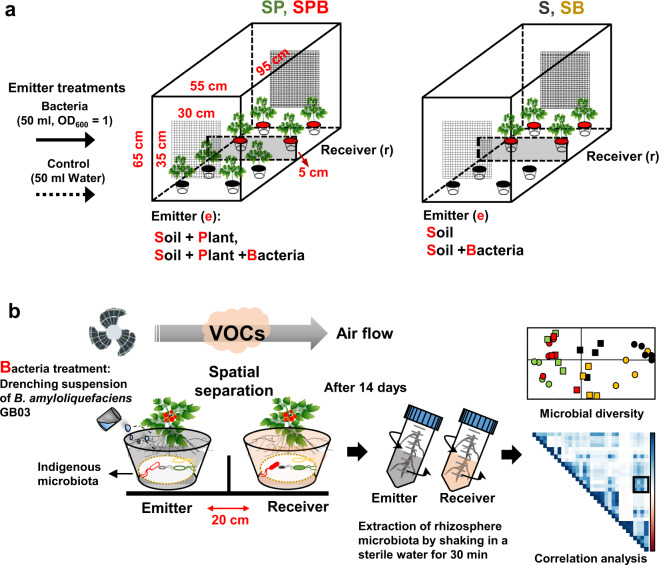
Experimental design on analysis of changes in the plant rhizosphere microbiome by microbe-induced plant. **a** Plot design. To confirm the interaction between emitter and receiver sectors through volatile organic compounds (VOCs), experiments were performed using plastic boxes in which below the soil signal transmission was blocked. Four plants were used per treatment. **b** Experimental steps for microbiome analysis. Plants were treated with *Bacillus amyloliquefaciens* strain GB03. Rhizosphere was sampled after 14 days and analysed using the MiSeq pipeline.

A dwarf cultivar of tomato (*S. lycopersicum* L.), Micro-Tom, was used in this study. Seeds of Micro-Tom were surface-sterilized with 3% sodium hypochlorite for 10 min and washed four times with sterile distilled water (SDW). The sterilized seeds were sown in pots (15 cm diameter) filled with autoclaved soil-less potting medium (Punong, Co. Ltd, Gyeongju, S. Korea) containing zeolite, perlite, coloured dust and lime (pH = 4.5–7.5). A miniature plastic box (55 cm wide × 95 cm long × 65 cm high) was built as a greenhouse simulation to grow tomato (emitter and receptor) plants, while protecting them from external air circulation. A 5-cm-tall partition was placed in the centre of the plastic box to prevent the mixing of root exudates between emitter and receptor treatments (Fig. 1a). Eight pots, each containing one Micro-Tom plant, were placed in the miniature box (four pots in the emitter (e) sector and four pots in the receiver (r) sector). Air flow from the emitter to the receiver sector was facilitated using an electric fan. Four treatments were performed in each sector: soil, soil + bacterium (*B. amyloliquefaciens* strain GB03), soil + plant and soil + plant + bacterium. These treatments were designated as eS, eSB, eSP and eSPB, respectively, in the emitter sector, and rS, rSB, rSP and rSPB, respectively, in the receiver sector. Four replicates were performed per treatment.

To conduct the eSB treatment, *B. amyloliquefaciens* strain GB03 was grown on tryptic soy agar (TSA; Difco Laboratories, Detroit, MI, USA) media at 30 °C for 1 day. The concentration of the PGPR culture was adjusted using SDW to an optical density (OD_600_) of 1.0. Micro-Tom plants treated with 50 mL SDW or drench application of *B. amyloliquefaciens* GB03 suspension (eS and eSB) were used as control emitter treatments (Fig. 1b).

### Amplicon sequencing and data analysis

After 14 days of GB03 treatment, Micro-Tom plants were explanted, and rhizosphere samples were analysed by 16S rRNA sequencing. DNA was extracted from the rhizosphere samples, and PCR amplification was performed using primers targeting regions V3 and V4 of the 16S rRNA gene. Bioanalyzer 2100 (Agilent, Palo Alto, CA, USA) with a DNA 7500 chip was used to check the quality of PCR products (as stated here) or to check the quality of the DNA isolated from rhizosphere samples. Amplicons were pooled and sequenced by Chunlab, Inc. (Seoul, S. Korea) using the Illumina MiSeq platform (Illumina, San Diego, USA), according to the manufacturer’s instructions.

Paired-end MiSeq reads were merged together using PANDAseq. Primer sequences were then trimmed using the Qiime programme (https://qiime2.org) at a similarity cut-off of 0.9. Non-specific amplicons (i.e., sequences that did not correspond to 16S rRNA) were detected using 16S rRNA profiles. Non-redundant reads were extracted using the UCLUST algorithm. Taxonomic assignment of sequences was performed based on the Ribosomal Database Project (RDP) using USEARCH (8.1.1861_i86linux32), followed by more precise pairwise alignment. UCHIME and the non-chimeric 16S rRNA database from RDP were used to detect chimeric reads with <97% best-hit similarity rate. Sequence data were then clustered using UCLUST. Alpha and beta diversity indices were estimated using the Qiime code.

### Analysis of VOCs released by emitter tomato plants

Plant VOCs were analysed by thermal desorption coupled with gas chromatography-mass spectrometry (TD–GC–MS). A GC-MS-QP 2010 Ultra gas chromatograph mass spectrometer (Shimadzu Corporation, Kyoto, Japan) equipped with an Rtx-5MS column [30 m length, 0.25 mm internal diameter (i.d.), 0.25 µm film thickness; Restek, USA] was used to perform TD–GC–MS. Polydimethylsiloxane (PDMS) tubing pieces were prepared as described previously [31]. Briefly, silicone tubing [1 mm i.d. × 1.8 mm outer diameter, Carl Roth GmbH, Germany] was cut into 0.5-cm pieces. The PDMS pieces were soaked in acetonitrile: methanol solution (4:1, v/v) overnight and baked for 3 h at 210 °C in glass columns under N_2_ gas flow. Then, the PDMS tubing pieces were cooled under N_2_ gas flow after purging argon gas. Each individual PDMS tubing piece was placed in 89-mm glass TD tubes (Supelco, USA) and desorbed under a stream of nitrogen (flow rate = 60 mL/min) at 200 °C for 8 min. All substances desorbed from the PDMS tubing piece were cryo-focused at −20 °C onto a Tenax® adsorbent trap in front of the column. After desorption, the Tenax® trap was heated to 230 °C within 10 s, and helium (He) split ratio of 1:20. Helium served as the carrier gas at a constant linear velocity of 40 cm/s. The TD–GC interface temperature was held at 230 °C. The GC column temperature was held at 40 °C for 5 min, then ramped up to 185 °C at a rate of 5 °C/min and increased further to 280 °C at a rate of 30 °C/min, where it was held for 0.83 min. The electron impact spectra were recorded at 70 eV in the scan mode at mass-to-charge ratios ranging from 33 to 400. The temperature of the transfer line and ion source was held at 240 °C and 220 °C, respectively. Data processing was performed using the GC–MS solution software (version 4.20; Shimadzu Corporation). The volatile compound β-caryophyllene was identified by matching the retention time and mass spectra of the sample with those of pure standards, while copaene and farnesol isomer were tentatively identified by matching the sample mass spectrum with the NIST14 library. The peak area of each compound was normalized by the peak area derived from the PDMS tubing piece at 15.5 min because this peak area was proportional to the PDMS tubing length [34].

### Analysis of phytohormones in root exudates

Micro-Tom seeds were surface-sterilized and germinated in vitro as described above. Four-day-old seedlings were transferred to Incu Tissue culture vessels (72 × 72 × 100 mm; SPL Life Sciences Co. Ltd, Pochen, S. Korea) containing 450 mL Murashige and Skoog liquid medium. The Incu Tissue culture vessels were placed in a glass jar (40-cm diameter, 60-cm height). Then, 0.01 mM β-caryophyllene (CAS no. 87-44-5; Sigma-Aldrich, Daejeon, S. Korea) was dispensed into plates (90-mm diameter) containing filter paper (5 × 5 cm). After 4 weeks of exposure to each emitter treatment or volatile chemicals, 20 mL root exudate was collected from the plate. The growth media was observed to ensure the absence of contamination. Then, the root exudate was extracted with 30 mL methanol containing an internal standard (SA-D4) and was subsequently evaporated. The residue was dissolved in 70% methanol, vortexed for 15 min and centrifuged at 15,000 rpm for 10 min at 4 °C. The supernatant was transferred to a glass vial for phytohormone analysis. This experiment was repeated three times.

Phytohormones were analysed by ultra-performance liquid chromatography coupled with quadrupole time-of-flight mass spectrometry (UPLC-QTOF-MS) using an ACQUITYVRUPLC system (Waters Corp., Milford, MA, USA) and a Q-TOF instrument (XEVO G2XS; Waters Corp.). Chromatographic separation was carried out on an ACQUITY UPLC BEH C18 column (100 × 2.1 mm, i.d., 1.7 μm) connected to an ACQUITY UPLC BEH C18 Van Guard pre-column (5 × 2.1 mm, i.d., 1.7 μm). The mobile phase consisted of solvent A (0.1% formic acid) and solvent B (acetonitrile). The gradient elution mode was programmed as follows: 5–60% B for 0.0–7.5 min and 60–95% B for 7.5–10.0 min. The column was then washed with 95% B for 3 min and equilibrated with 5% B for 2 min. All samples were maintained at 10 °C during the analysis. The flow rate and injection volume were 0.4 mL/min and 2 μL, respectively. MS analysis was conducted in the negative ion mode with electrospray ionization. Parameters used for MS were as follows: capillary voltage, 3 kV; cone voltage, 40 V; source temperature, 130 °C; desolvation temperature, 400 °C; cone gas flow, 50 L/h; and desolvation gas flow, 900 L/h. A calibration curve was constructed by plotting the ratio of peak area of the analyte to that of the internal standard. UPLC-QTOF-MS data were acquired and processed using the Masslynx software.

### Effect of SA on bacterial population dynamics

To assess the effect of methyl-SA treatment on the rhizosphere bacterial community, Micro-Tom plants were treated with 50 ng/mL methyl-SA (CAS no. 119-36-8; Sigma-Aldrich, St. Louis MO, USA) or SDW (control). Plant roots were collected at 4 weeks after treatment, transferred to a 30 mL conical tube (SPL Life Sciences, Pocheon, S. Korea) containing 10 mL SDW and shaken for 30 min. Root samples were placed on 1/10 strength TSA using a dilution plating method and cultured at 30 °C for 2 days. The number of bacterial colonies were counted to calculate the colony-forming units (cfu).

### Statistical analysis

Analysis of variance (ANOVA) of the experimental datasets was performed using JMP software (version 5.0; SAS Institute Inc., Cary, NC, USA; www.Sas.com) or R software (v3.6.3). Significant effects of treatments were determined based on the magnitude of the *F*-value (*P* < 0.05). When a significant *F*-value was obtained, separation of means was accomplished using the Fisher’s protected least square difference test at *P* = 0.05. Shannon index, inverse Simpson index, abundance-based coverage estimator and coverage were calculated using Qiime analysis. distance between samples was calculated using the UniFrac algorithm. Principal coordinate analysis was performed using the Bray–Curtis dissimilarity matrix. Correlation analysis was performed to confirm the similarity of microbial community between emitter and receiver treatments. Kendall, Pearson, and Spearman’s rank correlations were plotted using the “cor” and “corrplot” packages of the R software (v3.6.3). Microbial taxa and read numbers within emitter and receiver treatments were included in the analysis.

## Results and discussion

**Fig. 2:**
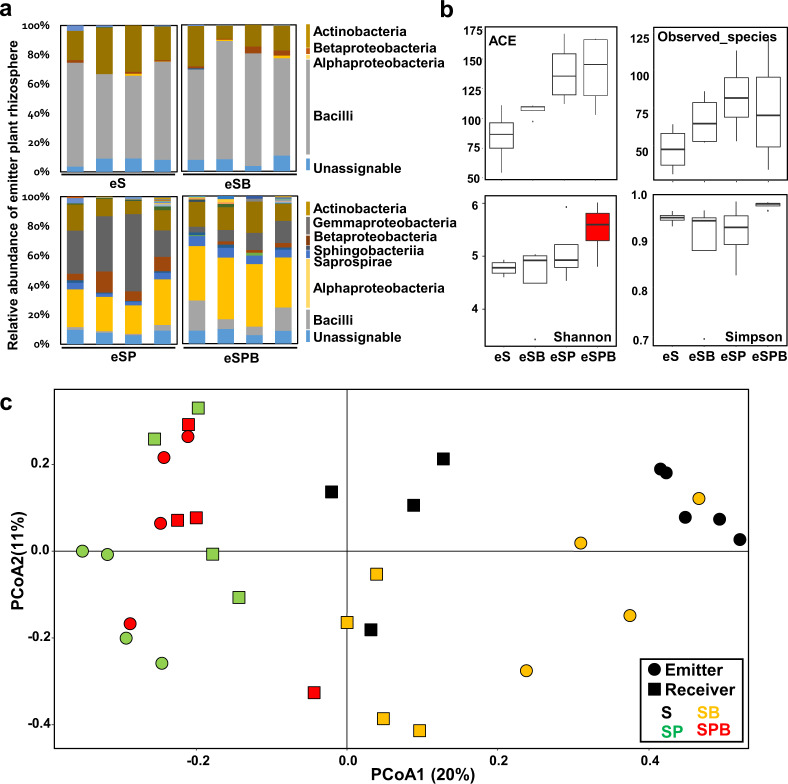
Microbiome analysis of emitter and receiver plant rhizosphere. **a** Relative abundance of using representative sequences at the 3% dissimilarity level **b** Alpha diversity analysis of the rhizosphere of emitter plants. Abundance-based coverage estimator (ACE) and observed species indicate richness diversity indices. Simpson and Shannon indicate evenness diversity indices. **c** Principal coordinate analysis (PCoA) performed using representative sequences at the 3% dissimilarity level. eS, eSB, eSP and eSPB represent emitter treatments; rS, rSB, rSP and rSPB represent receiver treatments. S, soil; SB, soil + bacterium; SP, soil + plant; SPB, soil + plant + bacterium.

To evaluate MIPV-mediated modulation of rhizosphere microbiota of a neighbouring plant as a proof of concept, we established a miniature greenhouse equipped with a fan to generate air-flow (Fig. 1) and evaluated changes in the rhizosphere microbiota of emitter tomato (cv. Micro-Tom) plants treated with PGPR. The results of pyrosequencing revealed that Firmicutes and Actinobacteria were the most abundant in the eS (emitter soil only) treatment, at 60% and 20%, respectively. In the eSP (emitter soil + plant) treatment, the proportion of Alpha-, Beta- and Gamma-proteobacteria increased significantly, while that of bacilli decreased to <10% (Fig. 2a). Compared with eSP, the eSPB (emitter soil + plant + bacteria (GB03) treated) treatment showed on average a 20% increase in bacilli, while the proportion of Gamma-proteobacteria decreased to 15% (Fig. 2a). The increase in bacilli in eSPB was possibly an effect of GB03. On the other hand, Gamma-proteobacteria are considered to be strongly associated with plants. Thus, physiological changes in plants triggered by GB03 can lead to an increase in the abundance of Gamma-proteobacteria in the rhizosphere (Fig. 2a). Chemical analysis of bacterial volatiles revealed the release of a series of low-molecular weight hydrocarbons including 2,3-butanediol [35]. Therefore, we used GB03-treated soil (eSB) as a control and compared it with eS to confirm the effect of bacterial volatiles on microbiota diversity. The results showed that bacterial volatiles did not affect the rhizosphere microbial community of the neighbouring plant (Fig. 2).

**Fig. 3:**
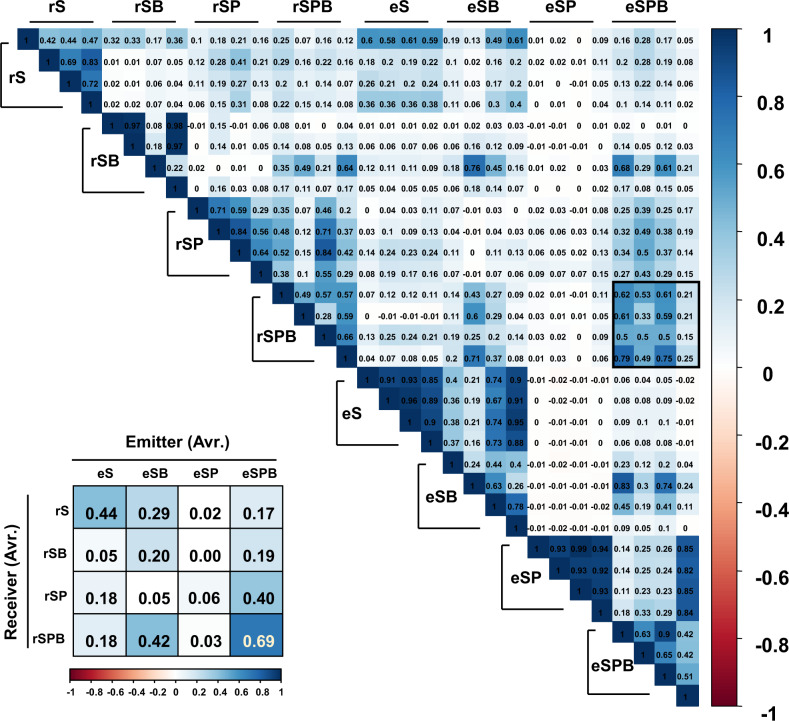
Determination of similarity in microbial communities between emitter and receiver sectors using Pearson’s correlation coefficient. The bold box in the right top pannel indicates in the raw data of Pearson’s correlation coefficient numbers for similar microbiota enrichment between the root system of PGPR-treated tomato plants and that of neighbouring tomato plants. The values of 16 box in the left bottom pannel indicate average of Pearson’s correlation coefficient numbers in each treatment. eS, eSB, eSP and eSPB represent emitter treatments; rS, rSB, rSP and rSPB represent receiver treatments. S, soil; SB, soil + bacterium; SP, soil + plant; SPB, soil + plant + bacterium.

The evenness of the rhizosphere microbial community was highest in the eSPB treatment (Fig. 2b). There was no statistical difference in microbial richness and evenness between treatments in the receiver section (Supplementary Fig. 1). High levels of evenness can indirectly increase the stability of microbial communities in the soil and protect them from plant diseases [28]. Beta diversity analysis showed that each treatment group was formed in the same manner as the emitter and receiver plants (Fig. 2c). This was supported by the analysis of microbial communities between the emitter and receiver treatments; Pearson’s correlation coefficient was highest (*r* = 0.69) between the eSPB and rSPB treatments, indicating that these two treatments were the most similar (Fig. 3). On the other hand, Kendall and Spearman correlations were not significant among various treatments (Supplementary Fig. 2). This suggests that Kendall and Spearman correlations are less sensitive than Pearson’s correlation coefficient. In addition, Pearson’s correlation analysis revealed similar microbiota enrichment between the root system of PGPR-treated tomato plants and that of neighbouring tomato plants. These results led us to investigate the determinant that modulates the rhizosphere microbiota of distant plants.

**Fig. 4:**
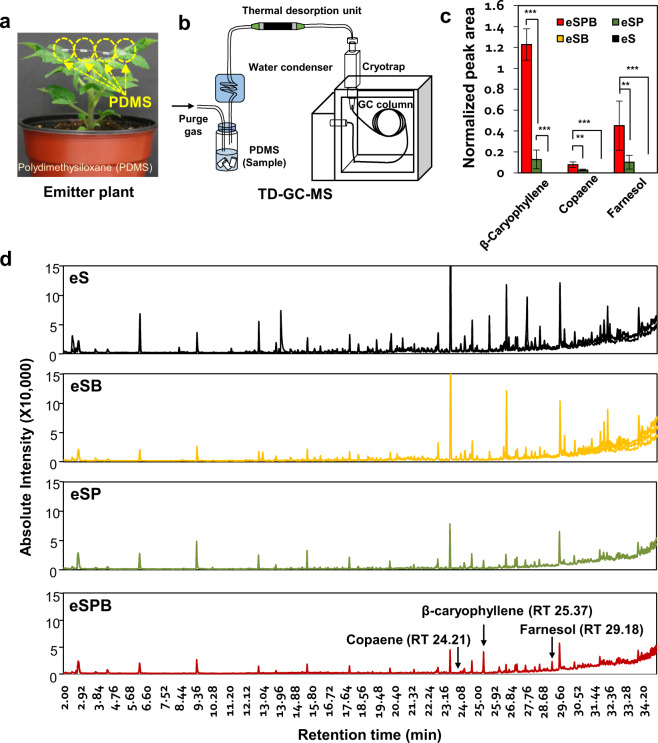
Profiling of plant VOCs from an emitter plant treated with PGPR. **a** VOCs released aboveground by emitter plants collected via polydimethylsiloxane (PDMS) tubing pieces. **b** Schematic of a TD–GC–MS system for plant volatile analysis. **c** Three eSPB-specific volatiles selected on the basis of peak area (normalized relative to the PDMS derived peak). **d** Total ion chromatograms of VOCs released by emitter plants. The experiment was performed in triplicate. Significant differences were determined by one-way analysis of variance (ANOVA), followed by Tukey´s honestly significant difference (HSD) test, and are indicated by asterisks (*P < 0.05, **P < 0.01, ***P < 0.001).

Out of several possible scenarios, we evaluated the MIPV-induced microbiota synchronizing theory [6], i.e., that VOCs released by tomato plants following root colonization by *B. amyloliquefaciens* strain GB03 (Fig. 4a) induce similar microbiota in neighbouring tomato plants. Three MIPVs, β-caryophyllene, copaene, and farnesol isomer, were uniquely detected in eSPB plants (Fig. 4b, c; Supplementary Fig. 3; Supplemenatary Table 1). Among these MIPVs, β-caryophyllene showed the highest peak area (normalized peak area, Fig. 4c) compared with other VOCs in the eSPB treatment (Fig. 4d). β-Caryophyllene is one of the 12 most common aromatic compounds of flowers [36]. Interestingly, β-caryophyllene is also involved in plant defence against pathogens [37]. The β-caryophyllene-rich rhizome oil of *Zingiber nimmonii* shows significant inhibitory activity against *B. amyloliquefaciens* and *Pseudomonas aeruginosa* [38]. (*E*) -β-cayophyllene, which is released from the flowers of *A. thaliana*, does not induce a defensive signalling pathway, but rather the pathogen *P. syringae* pv. tomato DC3000 directly inhibits the growth [37]. Farnesol, which was increased in eSPB treatment, is the precursor of steroids and sesquiterpenes, which are likely involved in the plant response to biotic stress. For example, farnesol is the precursor of farnese, which is an insect repellent in some plant species (such as those belonging to the family Solanaceae), and is involved, together with ethylene, in the onset of physiological disorder in apple fruit [39]. However, to date, little research has been conducted on changes in plant rhizosphere microbial communities caused by plant airborne signalling. Our results indicate that MIPV(s) produced in response to PGPR treatment can affect the rhizosphere microbial community of neighbouring plants, regardless of the distance between emitter and receiver plants.

**Fig. 5:**
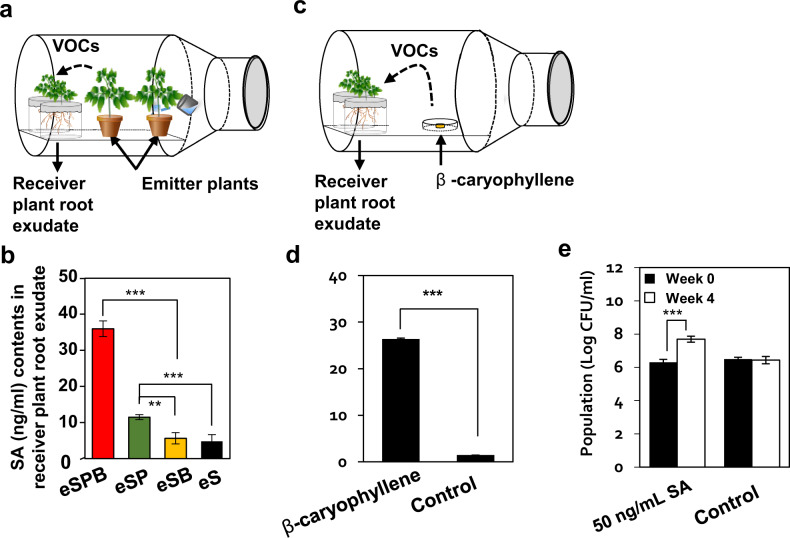
Profiling plant hormones in root exudates of receiver plant elicited by plant volatiles. **a** Analysis of phytohormone changes in receiver plants due to emitter treatment in glass containers. **b** Concentrations of salicylic acid (SA) in root exudates, as determined by ultra-performance liquid chromatography coupled with quadrupole time-of-flight mass spectrometry (UPLC-QTOF-MS). Average SA changes in receiver plants due to the release of plant volatiles by emitter plants. **c** Analysis of phytohormone changes in receiver plants due to β-caryophyllene treatment in glass containers. **d** Average SA changes in receiver plants due to the release of β-caryophyllene. **e** Total bacterial population in tomato plant rhizosphere measured at 0 and 4 weeks after challenge with 50 ng/mL MeSA in vitro. Water was used as a negative control. Experiments were performed in triplicate. Asterisks indicate significant differences (*P < 0.05, **P < 0.01, ***P < 0.001; one-way ANOVA, followed by Tukey’s HSD test).

Next, we questioned whether MIPVs induce physiological changes in receiver plants, leading to the secretion of root exudates and increasing the similarity of rhizosphere microbiota between emitter and receiver plants. To analyse the change in root exudates of the neighbouring (receiver) plant upon perceiving MIPVs from emitter plants, phytohormone analysis of the root exudate was performed by culturing the surrounding plants in a liquid medium under sterile conditions (Fig. 5a). The SA content of root exudates in the rSPB treatment was 36 ng/mL, which was significantly higher than that in other receiver treatments (rS, 4.8 ng/mL; rSB, 5.6 ng/mL; rSP; 11.5 ng/mL) (Fig. 5b; Supplementary Fig. 4). The amount of JA and abscisic acid (ABA) in root exudates showed no significant differences among the four treatments (Supplementary Figs. 5, 6 and 7). We did not detect a significant difference between emitter and receiver treatments, except in the amount of a plant hormone, although we attempted to assess various root exudate candidates (data not shown).

To confirm the effect of changes in phytohormone levels on tomato root exudates by the pharmaceutical application of the signature MIPV β-caryophyllene, tomato seedlings were exposed to β-caryophyllene in a closed glass jar (Fig. 5c). The SA content in the root exudates of receiver plants was 27 ng/mg (Fig. 5d; Supplementary Fig. 8). However, other plant hormones, including JA and ABA, were undetectable (data not shown). Invaded plants use volatile substances as signals to rapidly transmit their status to surrounding plants and to induce the surrounding plants to trigger an immune response [11, 18, 40]. Nonanal, a plant volatile, reduced the population density of *P. syringae* pv. syringae, and consequently disease severity, in neighbouring plants when systemic acquired resistance (SAR) elicited by an avirulent pathogen occurred in emitter lima bean plants [15]. In *Arabidopsis*, exposure to monoterpenes not only increases the production of reactive oxygen species but also induces SAR through SA signalling [41, 42]. Additionally, resistance to the fungal pathogen *Colletotrichum lindemuthianum* in susceptible varieties increased plant immunity when exposed to VOCs released by resistant varieties for 6 h. The susceptible common bean (*Phaseolus vulgaris*) cultivar was primed resistance marker genes (*Pathogenesis-related 1* (*PR1*)*, PR2*, and *PR4*) by exposed to VOCs of the resistant cultivar against a fungal pathogen [14].

 Plant volatiles are used for communication with organisms such as insects, nematodes, bacteria, fungi and viruses [5, 20]. Under biotic and abiotic stresses, changes in phytohormone levels in the root exudate affect the plant rhizosphere microbial community [24, 25]. Our results confirmed that the microbial density increased to 10^8^ cfu/mL in the rhizosphere of tomato plants treated with 50 ng/mL SA, and to 10^6^ cfu/mL in the control treatment (Fig. 5e). The interaction between plant and microbial communities is very complex and dynamic. The plant immune system is thought to play an important role in determining the structure of plant rhizosphere microbiome [43]. Differences were observed in the rhizosphere bacterial community composition of *Arabidopsis* mutants defective in SAR and that of wild-type plants [25]. *Arabidopsis* mutants with a disrupted JA pathway showed increased abundance of *Streptomyces*, *Bacillus*, Enterobacteriaceae and *Lysinibacillus* taxa in the rhizosphere [24]. Recently, it has also been shown that VOCs released from the roots of *Carex arenaria* plants infected with *Fusarium culmorum* can stimulate long-range soil migration of certain bacteria with antifungal properties [44].

However, the role and mechanisms of volatiles in the plant rhizosphere microbiome remain largely unknown. Our results confirm that VOCs released aboveground (headspace) by biologically stimulated plants affect the rhizosphere microbiota of surrounding plants by regulating the phytohormone contents of their root exudates. No bacterial volatiles were detected in the headspace (data not shown). Overall, our study highlights the role that plant MIPVs play as long-distance signals in achieving similar root microbiota composition of spatially separated emitter and receiver plants. However, it is not possible to rule out the possibility that the composition of other volatiles, not just the specific volatiles identified in our study, will affect the surrounding plants in combination.

**Fig. 6:**
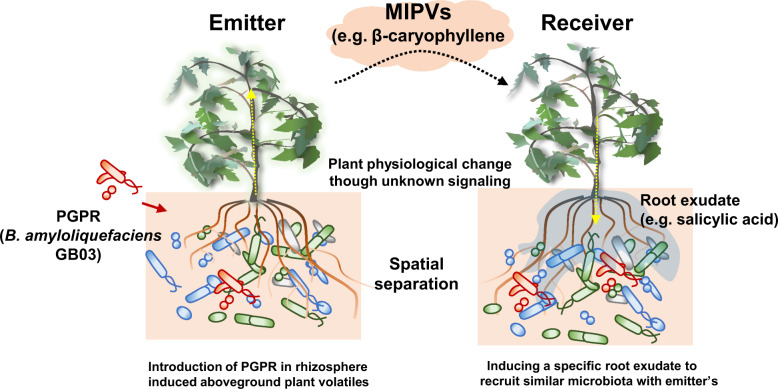
Schematic representation showing the build-up of microbial community through the production of microbe-induced plant volatiles (MIPVs) and the reaction of surrounding plants, along with the increase in SA in the root exudate. The reaction of surrounding plants that have undergone volatile signalling changes in the composition of SA in plant root secretions, affecting microorganisms and stimulating specific microbial dominance, similar to the microbial community of emitter plants.

Previous studies on various ecological systems suggest that volatiles released by a plant affect the defence mechanisms of distant plants [45]. However, the effects of MIPVs on plant and microbial ecology are largely unknown. Our results firstly demonstrate a potential mechanism that explains how the rhizosphere microbial community of distant plants can be synchronized through airborne signalling (Fig. 6). Volatile substances can act as aerial signals to facilitate plant–plant interactions within a distance of 1 m [5]. Our findings can be used to develop a novel plant fitness modulator for reducing plant disease and promoting plant growth with PGPR treatment. However, a possible pitfall of this study is the use of PGPR to induce MIPV emission since the low survival capacity of the introduced bacteria under natural conditions. Nonetheless, we believe that this study provides a novel means to engineer rhizosphere microbiota through the application of specific MIPVs and root exudates to create an ideal soil microbiome that improves plant health by decreasing plant disease incidence and promoting plant growth and yield.

Although we described a new phenomenon driven by an MIPV, this study addresses only the tip of the iceberg. Several questions remain unanswered. How do *B. amyloliquefaciens* strain GB03 and bacterial determinants modulate rhizosphere microbiota of the emitter plant? What is the maximum distance that β-caryophyllene can travel to affect the receiver plant? What acts as the β-caryophyllene receptor in tomato? Are there any other new determinants of root exudates and MIPVs that modulate rhizosphere microbiota? How does SA affect rhizosphere microbiota? Finding the answers to these questions will facilitate the development of more sophisticated tools that could be used to manage plant fitness and rhizosphere microbiota. Collectively, our current findings re-confirm our previous suggestion that plants and microbes should be developed together, not separately.

The total biome of plants and microbes has recently been recognized as a ‘holobiont’ and has been studied for interrelationship for a long time. Volatile-mediated communication acts as a universal language in the plant and microbe interaction [5, 46]. MIPVs might contribute to the propagation of defence-associated microbiota from one plant to another in agricultural environments. Therefore, creating a healthy soil microbiome using interplant volatile signals will help to minimize the application of chemicals, thus enabling the development of sustainable agriculture. To modify rhizosphere microbiota in the natural environment using MIPVs, it is important to determine the effective distance of volatile emissions, methods of VOC encapsulation and the effect of VOCs on other plant species.

## Supplementary information

Achieving similar root microbiota composition in neighbouring plants through airborne signalling
