# Supplementary material for: Achieving similar root microbiota composition in neighbouring plants through airborne signalling
Source: ISME J. 2020 Sep 24;15(2):397–408. doi: 10.1038/s41396-020-00759-z (PMC8027813; doi:10.1038/s41396-020-00759-z)
Supplement: Supplementary file 1 — Achieving similar root microbiota composition in neighbouring plants through airborne signalling [file 41396_2020_759_MOESM1_ESM.docx]

**Achieving similar root microbiota composition in neighbouring plants through airborne signalling**

Hyun Gi Kong^1,2^, Geun Cheol Song^1,3^, Hee-Jung Sim^4^, and Choong-Min Ryu^1,*^

^1^Molecular Phytobacteriology Laboratory, Infectious Disease Research Center, KRIBB, Daejeon 34141, South Korea

^2^Crop Protection Division, National Institute of Agricultural Sciences, Rural Development Administration, Wanju-gun, 54875, South Korea

^3^Plant Nutrition Laboratory, Industrial Microbiology Center, CJ, Suwon 16509, South Korea ^4^Environmental Chemistry Research Group, Korea Institute of Toxicology (KIT), 17 Jegok-gil, Munsan-eup, Jinju 52834, South Korea

**Running title:** MIPVs synchronize root microbiota

**^*^Corresponding author:**

Choong-Min Ryu

Tel.: +82-42-879-8229

Fax: +82-42-860-4488

E-mail: [cmryu@kribb.re.kr](mailto:cmryu@kribb.re.kr)

**Supplementary Figure**

**
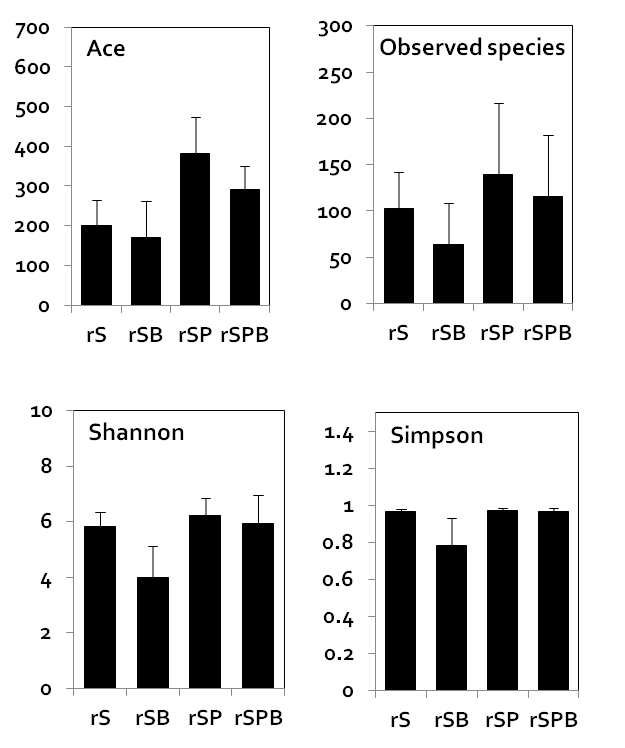
**

**Supplementary Fig. 1** Alpha diversity analysis of receiver plants using representative sequences at the 3% dissimilarity level. Abundance-based coverage estimator (ACE) and observed species indicate richness diversity indices. Simpson and Shannon indicate evenness diversity indices.

**
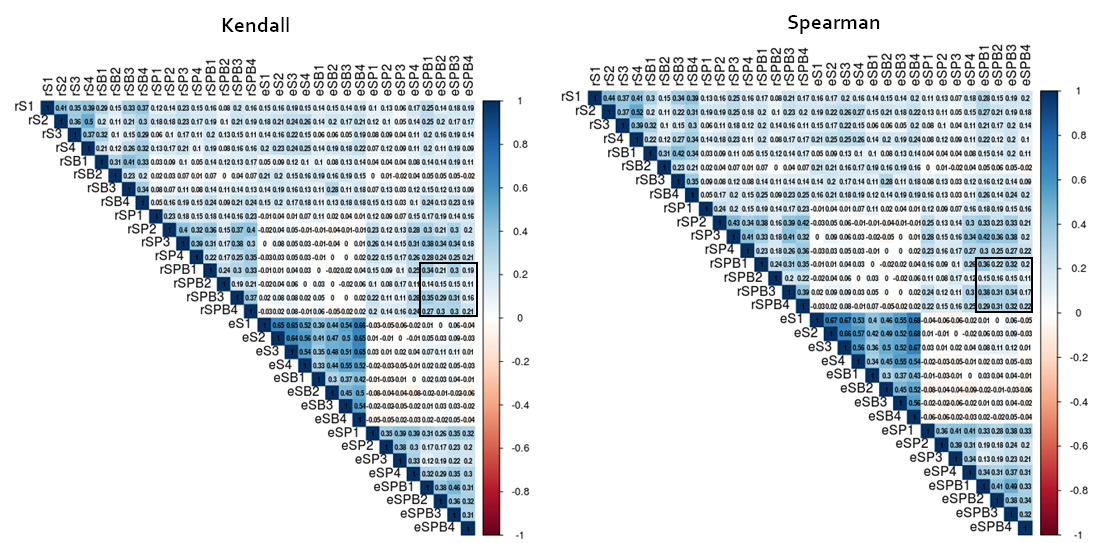
**

**Supplementary Fig. 2** Determination of similarity in microbial communities between emitter and receiver sectors using Kendall and Spearman correlations and Pearson's correlation coefficient. Four plants were used per treatment.

**
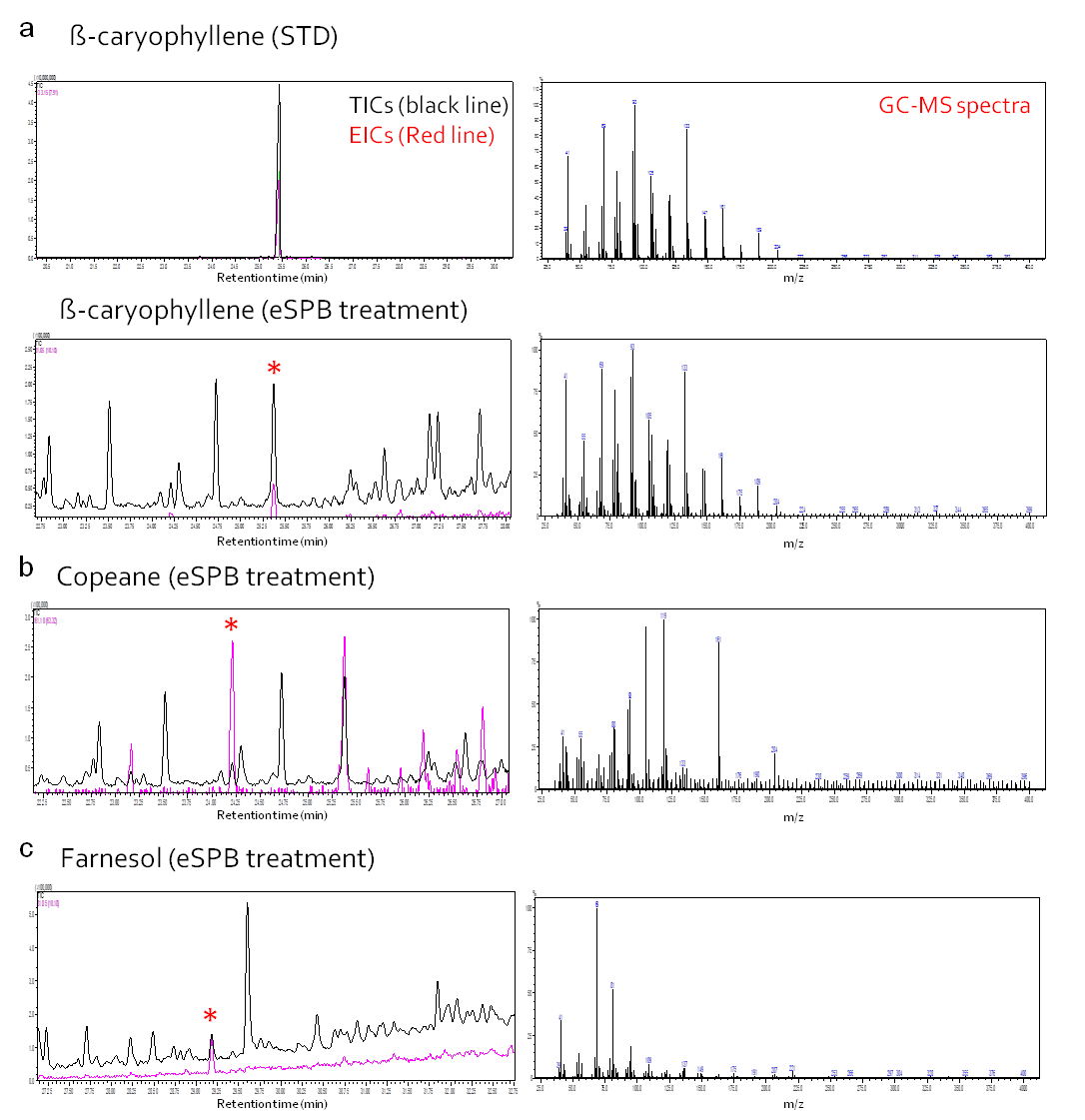
**

**Supplementary Fig. 3** The EICs (red line) and MS spectra of Gas chromatography-mass spectrometry (GC-MS) for the identification of (a) beta-caryophyllene in eSPB treatment and standard compound (peaks indicated by asterisk). Represents EICs and MS spectra of copeane (b) and Farnesol (c) in eSPB.

**
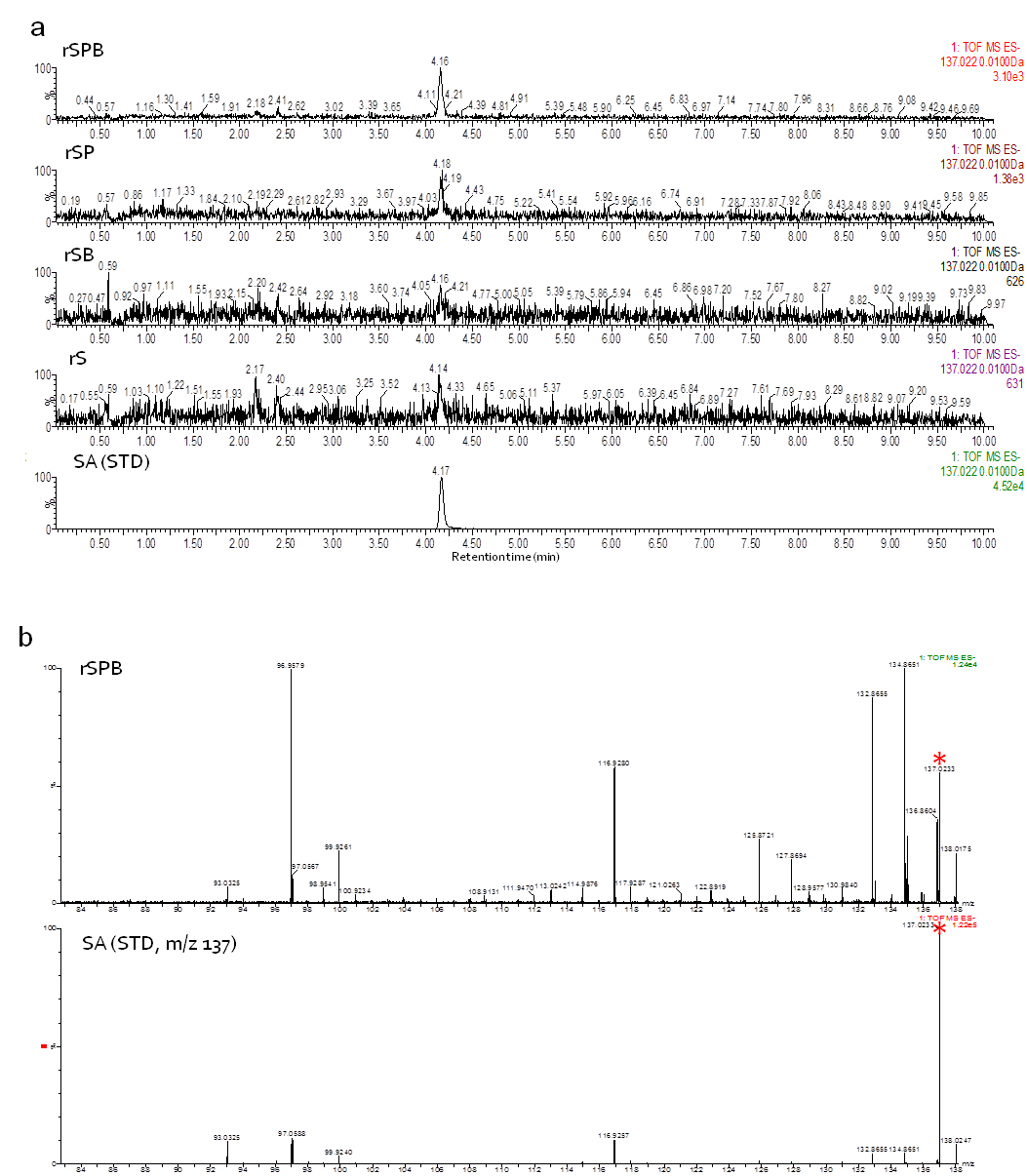
**

**Supplementary Fig. 4** The EICs (a) and MS spectra (b) of liquid chromatography-mass spectrometry (LC-MS) for the identification of salicylic acid (SA, m/z 116) in root exudates of receptor treatments and standard compound. The SA concentration of the rSP, rSB and rS treatments was below the limit of quantitation (LOQ).

**
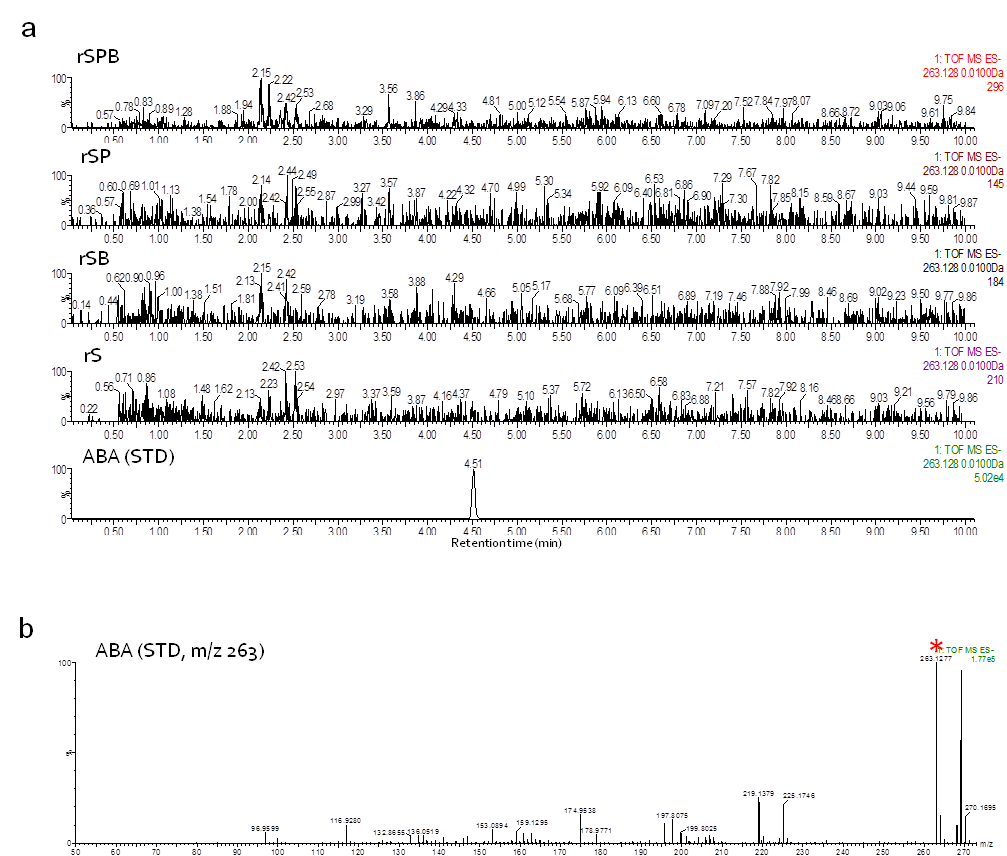
**

**Supplementary Fig. 5** The EICs (a) and MS spectra (b) of liquid chromatography-mass spectrometry (LC-MS) for the identification of abscisic acid (ABA, m/z 263) in root exudates of receptor treatments and standard compound. The SA concentration of the all treatments was below the limit of quantitation (LOQ).

**
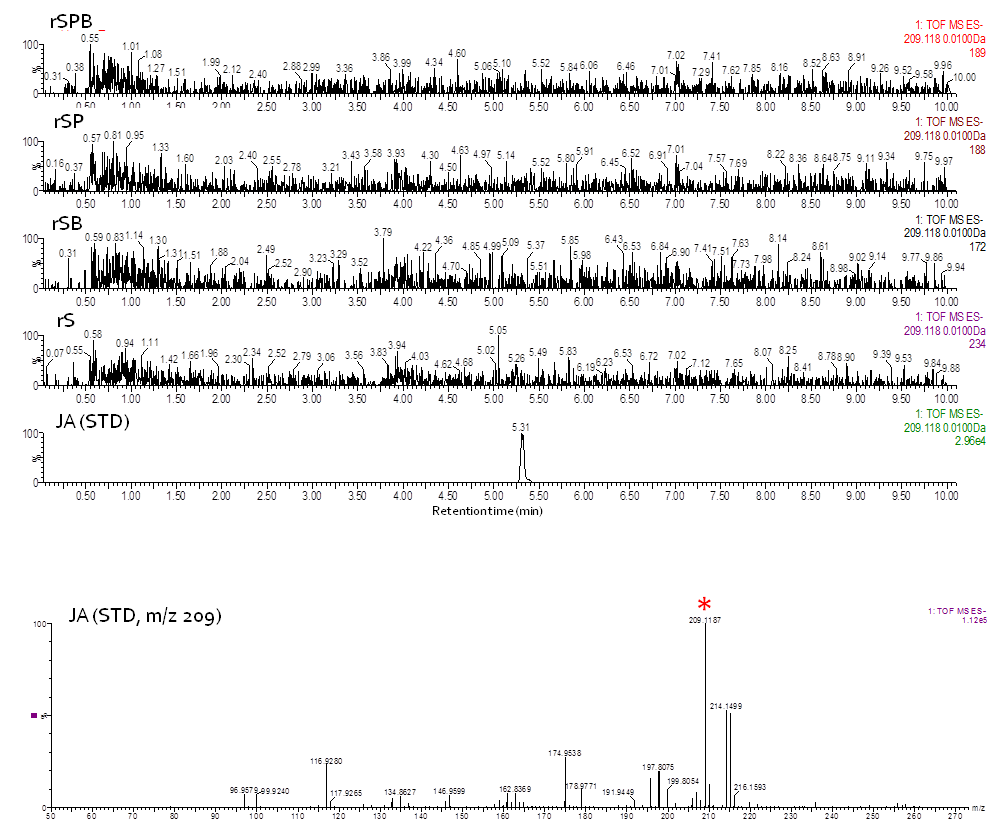
**

**Supplementary Fig. 6** The EICs (a) and MS spectra (b) of liquid chromatography-mass spectrometry (LC-MS) for the identification of jasmonic acid (JA, m/z 209) in root exudates of receptor treatments and standard compound. The JA concentration of the all treatments was below the limit of quantitation (LOQ).

**
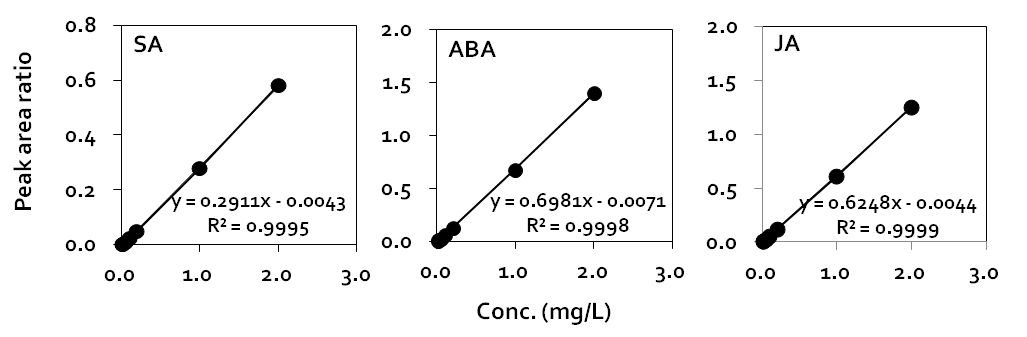
**

**Supplementary Fig. 7** Concentrations of jasmonic acid (JA) and abscisic acid (ABA) in root exudates, and calibration curves of salicylic acid (SA), JA and ABA. **a** Calibration curves of SA, JA and ABA constructed using seven different concentrations of each phytohormone. **b** Concentrations of JA and ABA in root exudates, as determined by ultra-performance liquid chromatography coupled with quadrupole time-of-flight mass spectrometry (UPLC-QTOF-MS).

**
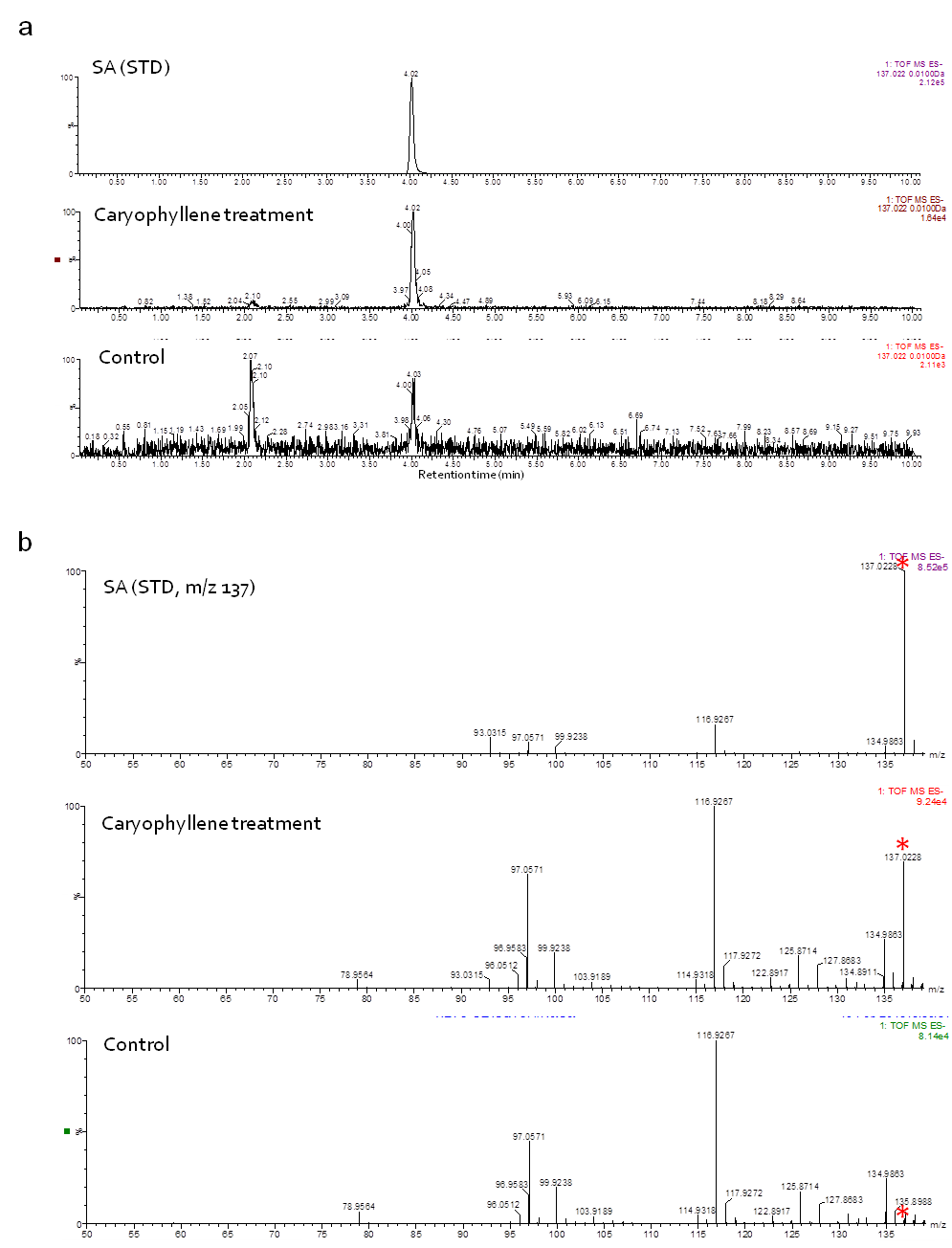
**

**Supplementary Fig. 8** The EICs (a) and MS spectra (b) of liquid chromatography-mass spectrometry (LC-MS) for the identification of salicylic acid (SA, m/z 116) in beta-caryophyllene treatments, control and standard compound (peaks indicated by asterisk). The SA concentration of the control treatment was below the limit of quantitation (LOQ).

**Supplementary Table**

**Supplementary Table 1.** GC/MS Chromatogram compound list of emitter plants

| **Retention time** | **Name of the compound** | **Treatments** | **Molecular formula** |
| --- | --- | --- | --- |
| 9.415 | Methyl N-hydroxybenzenecarboximidoate | eS, eSB, eSP, eSPB | C8H9NO2 |
| 13.81 | 2-Ethyl-1-hexanol | eS, eSB, eSP, eSPB | C8H18O |
| 16.275 | Nonanal | eS, eSB, eSP, eSPB | C9H18O |
| 17.205 | Acetic acid, 4-hydroxy-cyclohexyl ester | eS, eSB, eSP, eSPB | C8H14O3 |
| 18.4 | 7-Oxooctanoic acid | eS, eSB, eSP, eSPB | C8H14O3 |
| 18.93 | 1-(2-Butoxyethoxy)ethanol | eS, eSB, eSP, eSPB | C8H18O3 |
| 19.02 | 2-Decanone | eS, eSB, eSP, eSPB | C10H20O |
| 19.635 | 2-Ethyl-2-propyl-1-hexanol | eS, eSB, eSP, eSPB | C11H24O |
| 20.54 | 1-Monoacetin | eS, eSB, eSP, eSPB | C5H10O4 |
| 20.945 | 4-Hydroxyoctanoic acid lactone | eS, eSB, eSP, eSPB | C8H14O2 |
| 21.115 | Methyl 2-ethyl-2-methylpentanoate | eS, eSB, eSP, eSPB | C9H18O2 |
| 21.175 | Neodecanoic acid | eS, eSB, eSP, eSPB | C10H20O2 |
| 21.215 | Tridecanoic acid | eS, eSB, eSP, eSPB | C13H26O2 |
| 21.32 | 5-Undecanone | eS, eSB, eSP, eSPB | C11H22O |
| 21.5 | 2,6,11-Trimethyldodecane | eS, eSB, eSP, eSPB | C15H32 |
| 21.95 | 2-Tridecanone | eS, eSB, eSP, eSPB | C13H26O |
| 21.955 | cis-Hexahydrophthalide | eS, eSB, eSP, eSPB | C8H12O2 |
| 22.055 | Oxalic acid, 2-ethylhexyl isohexyl ester | eSPB | C16H30O4 |
| 22.235 | cis-3-Hexadecene | eSPB | C16H32 |
| 22.465 | Trichloroacetic acid, pentadecyl ester | eSPB | C17H31Cl3O2 |
| 22.83 | 1-Ethylpropyl 2-ethylhexanoate | eS, eSB, eSP, eSPB | C13H26O2 |
| 23.51 | 1,2-Diacetin | eS, eSB, eSP, eSPB | C7H12O5 |
| 24.08 | 3-Hydroxy-2,2,4-trimethylpentyl 2-methylpropanoate | eS, eSB, eSP, eSPB | C12H24O3 |
| 24.21 | Copaene | eSPB | C15H24 |
| 24.3 | 5-(Hydroxymethyl)-3,3-dimethyldihydro-2(3H)-furanone | eS, eSB, eSP, eSPB | C7H12O3 |
| 24.72 | Tetradecane | eS, eSB, eSP, eSPB | C14H30 |
| 25.37 | Caryophyllene | eSPB | C15H24 |
| 25.68 | 1,4-Diacetylbenzene | eS | C10H10O2 |
| 26.62 | 1-Dodecanol | eS, eSB, eSP, eSPB | C12H26O |
| 27.13 | Eicosane | eSP, eSPB | C20H42 |
| 27.23 | Eicosyl isopropyl ether | eSP, eSPB | C23H48O |
| 27.7 | 2,4-Dichlorobenzoic acid | eS, eSB, eSP, eSPB | C7H4Cl2O2 |
| 28.015 | Acetic acid, trifluoro-, dodecyl ester | eS, eSB, eSP, eSPB | C14H25F3O2 |
| 28.22 | 2-Methylhexacosane | eSP, eSPB | C27H56 |
| 28.48 | Docosanoic acid, docosyl ester | eSP, eSPB | C44H88O2 |
| 28.73 | 2-Methyltetracosane | eS, eSB, eSP, eSPB | C25H52 |
| 29.18 | Farnesol isomer a | eSPB | C15H26O |
| 29.6 | 2-methyloctacosane | eS, eSB, eSP, eSPB | C29H60 |
| 29.85 | n-Hexadecyl ethanoate | eS, eSB, eSP, eSPB | C18H36O2 |
| 30.42 | 10-12-Pentacosadiynoic acid | eS, eSB, eSP, eSPB | C25H42O2 |
| 30.695 | 3,5,24-Trimethyltetracontane | eS, eSB, eSP, eSPB | C43H88 |
| 30.775 | 2-Dodecen-1-yl(-)succinic anhydride | eS, eSB, eSP, eSPB | C16H26O3 |
| 31.84 | 2-Methylhexacosane | eSP, eSPB | C27H56 |
| 32.05 | Eicosanoic acid, 2,3-bis(acetyloxy)propyl ester | eS | C27H50O6 |
| 32.25 | 2-Tetradecyloxyethanol | eS, eSB | C16H34O2 |
| 33.98 | 2-Methyltetracosane | eS, eSB, eSP, eSPB | C25H52 |
